# The Roles of FoxO Transcription Factors in Regulation of Bone Cells Function

**DOI:** 10.3390/ijms21030692

**Published:** 2020-01-21

**Authors:** Xiaoli Ma, Peihong Su, Chong Yin, Xiao Lin, Xue Wang, Yongguang Gao, Suryaji Patil, Abdul Rouf War, Abdul Qadir, Ye Tian, Airong Qian

**Affiliations:** 1 Lab for Bone Metabolism, Key Lab for Space Biosciences and Biotechnology, School of Life Sciences, Northwestern Polytechnical University, Xi’an 710072, China; xiaoli225@mail.nwpu.edu.cn (X.M.); suph@mail.nwpu.edu.cn (P.S.); yinchong42@mail.nwpu.edu.cn (C.Y.); linxiao@nwpu.edu.cn (X.L.); wangxue1005@mail.nwpu.edu.cn (X.W.); gaoyongguang@nwpu.edu.cn (Y.G.); suryajip@mail.nwpu.edu.cn (S.P.); roufbio_2017@mail.nwpu.edu.cn (A.R.W.); abdulqadirwazir145@yahoo.com (A.Q.); 2 Research Center for Special Medicine and Health Systems Engineering, School of Life Sciences, Northwestern Polytechnical University, Xi’an 710072, China; 3 NPU-UAB Joint Laboratory for Bone Metabolism, School of Life Sciences, Northwestern Polytechnical University, Xi’an 710072, China

**Keywords:** FoxOs, bone cells, antioxidant, osteogenesis, osteoclastogenesis, chondrogenesis, bone diseases

## Abstract

Forkhead box class O family member proteins (FoxOs) are evolutionarily conserved transcription factors for their highly conserved DNA-binding domain. In mammalian species, all the four FoxO members, FoxO1, FoxO3, FoxO4, and FoxO6, are expressed in different organs. In bone, the first three members are extensively expressed and more studied. Bone development, remodeling, and homeostasis are all regulated by multiple cell lineages, including osteoprogenitor cells, chondrocytes, osteoblasts, osteocytes, osteoclast progenitors, osteoclasts, and the intercellular signaling among these bone cells. The disordered FoxOs function in these bone cells contribute to osteoarthritis, osteoporosis, or other bone diseases. Here, we review the current literature of FoxOs for their roles in bone cells, focusing on helping researchers to develop new therapeutic approaches and prevent or treat the related bone diseases.

## 1. Introduction

Forkhead box class O family member proteins (FoxOs) are evolutionarily conserved transcription factors [1]. FoxOs translate environmental signaling into gene expression [2], regulating many cellular processes, including cell survival, proliferation, differentiation, apoptosis, oxidative stress resistance, metabolism, inflammation, and aging [3,4,5,6,7,8]. In bone, internal and external environmental changes cause an imbalance of the bone dynamic, resulting in loss of bone mass and strength, and increased risk for fractures and morbidity [9,10]. Recent studies with murine models of cell-specific loss- or gain-of-function of FoxOs, have revealed that FoxOs regulate bone cell functions and their intercellular signaling. These controls affect bone development, alter bone mass, and conduce several bone diseases, such as osteoarthritis, age-related or estrogen-deficiency osteoporosis, and so on [10].

Hence, we will firstly introduce the structure and function of the FoxO family. Then, we will review the recent developments about FoxOs in osteoprogenitor cells, osteoblasts, hematopoietic stem cells, osteoclast progenitors, osteoclasts, and chondrocytes, as well as the intercellular signaling among these bone cells. At the same time, we will also describe the FoxO actions in bone cell biology, including cell survival, self-renewal, cell cycle, proliferation, osteogenesis, osteoclastogenesis, chondrogenesis, autophagy, and apoptosis. All of these will focus on their emerging molecular mechanism, pathological implications, and therapeutic potential for related bone diseases.

## 2. The FoxO Family

**Figure 1:**
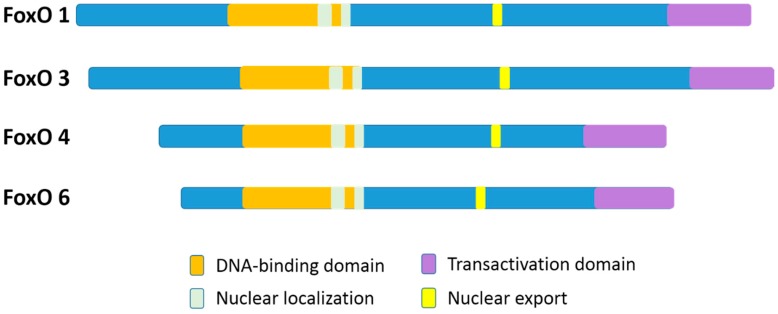
A schematic diagram of the four different functional motifs of the mammalian FoxO family members (FoxOs). For humans, FoxO1, FoxO3, FoxO4, and FoxO6 proteins are 655 aa, 673 aa, 505 aa, and 492 aa in size, respectively. The functional domains are the forkhead domain, nuclear localization, nuclear export, and a transactivation domain.

In mammalian species, there are four members of the FoxO family: FoxO1 (FKHR), FoxO3 (FKHRL1), FoxO4 (AFX), and FoxO6 [11]. FoxO1 and FoxO3 proteins are greater than 650 amino acids in size, which are larger than FoxO4 and FoxO6 (nearly 500 amino acids). Although, the FoxO6 gene exhibits major structural differences as compared to the other three family gene members [11]. The four FoxO protein members share obvious sequence homology and possess four clearly different functional motifs, which include a forkhead domain, nuclear localization, nuclear export, and transactivation domains (Figure 1) [12,13]. All the four FoxO members are ubiquitously expressed [14], including FoxO6 that was previously reported to exist basically in the brain but has been more recently discovered in other organs as well [4,15]. In particular, FoxO1, FoxO4, and FoxO6 are more detected in the adipose, musculoskeletal, and nervous tissues, respectively, while FoxO3 is more expressed in the stomach, spleen, kidney, intestine, and cardiac tissues [13]. Nevertheless, FoxO1, FoxO3, and FoxO4 are all expressed in bone cells [16].

In FoxO proteins, the forkhead domain located in the N-terminal portion [17] can recognize two response elements: the insulin-responsive element (5’-(C/A) (A/C)AAA(C/T)AA) and Daf-16 (namely FoxO) family member binding element (5´-GTAAA(T/C)AA) [12,13,14,15,16,18]. Although FoxO1 recognizes both the insulin response element and the Daf-16 family member binding element, it has a higher affinity for the latter [19]. The transactivation domain, located in the C-terminal region of the FoxOs, combines with the cis-regulatory sites of FoxO-target genes [17]. In addition, the nuclear localization sequence and the nuclear export sequence are within the C-terminal DNA binding domain of FoxOs, indispensable for maintaining the FoxO proteins in the nucleus or in the cytosol, respectively [12]. Nuclear FoxO proteins, predominantly as transcription factors, interact with DNA and partner proteins to modulate the transcription of numerously specific target genes [17]. 

Responding to a variety of external or internal stimuli (including starvation, insulin, growth factors, hormones, cytokines, and oxidative stress), FoxOs act as transcription factors and rarely as co-regulators, either activated or inactivated by post-translational modification (like phosphorylation, ubiquitination, acetylation, and methylation) [9,20]. Further, they are also regulated by lipopolysaccharide (LPS), tumor necrosis factor α (TNF-α), and the interaction with protein partners [21]. These modifications alter the nuclear import and export steps of FoxOs, modify the DNA binding affinity of FoxOs, and alter transcriptional activity for FoxOs’ specific target gene. Therefore, FoxOs take part in regulatory networks that ensure tight and timely transcriptional control of proteins involved in proliferation (FoxO1, FoxO3, and FoxO4), cellular differentiation (FoxO1, FoxO3, and FoxO4), apoptosis (FoxO1, FoxO3, and FoxO4), oxidative stress resistance (FoxO1 and FoxO3), metabolism (FoxO1 and FoxO3), inflammation (FoxO1, FoxO3, and FoxO4) and aging (FoxO1, FoxO3, and FoxO4) in mammals [3,4,5,6,7,8]. What is more, a unifying hypothesis has also highlighted the role of FoxOs as signaling integrators for the maintenance of cell and tissue homeostasis over time and in response to environmental challenges, including metabolic stress, oxidative stress, and growth factor deprivation [9].

## 3. FoxOs and Bone Cells

As compared to other organs in the body, bone is constantly degraded and replaced throughout one’s life. The relatively dynamic bone modeling and remodeling are governed specifically by osteoprogenitor cells, osteoblasts, osteocytes, hematopoietic stem cells, osteoclast progenitors, osteoclasts, and chondrocytes, as well as the complex intercellular signaling among these bone cells. Except for osteocytes, all bone cells were found expressing FoxO1, 3, and 4, as in several other mammalian cell types. Functions of FoxOs in these cells are pivotal for bone development, remodeling, and homeostasis manipulation under physiological and pathological conditions.

### 3.1. Bidirectional Regulation of FoxOs in Osteoprogenitor Cells

FoxOs play important roles in the osteogenesis of osteoprogenitor cells containing mesenchymal stem cells (MSCs), the early precursors of osteoblasts. MSCs are multipotent stromal cells. They give rise to chondrocytes, muscle cells, and adipocytes. Moreover, osteogenic differentiation of MSCs into osteoblasts is managed by an array of specific transcription factors (such as transcription factor TCF, runt-related transcription factor 2, and activating transcription factor 4) and genes (such as the alkaline phosphatase gene, runt-related transcription factor 2 gene, and osteocalcin gene), leading to a succession of osteoblast phenotypic markers [16]. Therefore, it is not difficult to envision the bidirectional regulation role of FoxOs in the entire complex and sensitive osteogenic process (Table 1).

#### 3.1.1. FoxOs Promote Osteogenesis

**Figure 2:**
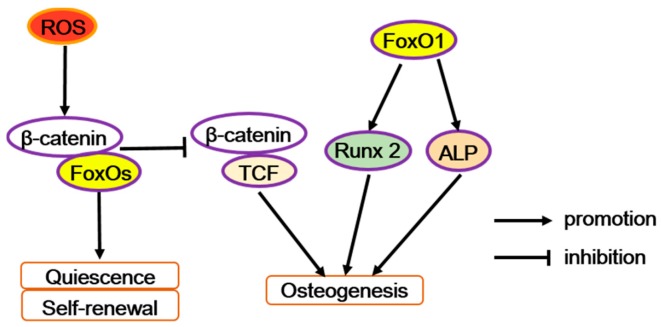
The diverse function of FoxOs in osteoprogenitor cells. Reactive oxygen species (ROS) can trigger FoxOs mediated transcription and enhances the binding of FoxOs to β-catenin, thus diverting the limited β-catenin pool from transcription factor TCF to FoxO-mediated transcription and thereby decreases osteoblastogenesis. But FoxO1 promotes the transcription of runt-related transcription factor 2 (*Runx2*) or alkaline phosphatase (*Alp*), increasing osteoblastogenesis.

With experiments in vitro, ex vivo, and in vivo, Teixeira et al. have demonstrated FoxO1 as an early positive regulator in the osteogenic differentiation of mesenchymal cells [22]. In mouse embryonic mesenchymal cells (C3H10T1/2 cells), osteogenic stimulants enhance the activity and expression of FoxO1. Similarly, overexpressing FoxO1 significantly increases the expression of osteogenic markers such as runt-related transcription factor 2 (*Runx2*), alkaline phosphatase (*Alp*), and osteocalcin (*Ocn*). Conversely, silencing FoxO1 inhibits the expression of these osteogenic markers, decreases calcification, impairs skeletogenesis and craniofacial development, and especially reduces the size of the embryos as well as bone size in the craniofacial area. In addition, knocking down FoxO1 in mouse embryonic tibia ex vivo has been reported to have similar results as those obtained in vivo, which led to shorter and less mineralized bone [22]. Furthermore, in pre-osteoblastic cells (e.g., MC3T3-E1 cells), the upregulated binding activity of FoxO1 to the Runx2 gene promoter increases the mRNA level of *Runx2* and alkaline phosphatase (ALP) activity during the formation of mineralizing nodules. In addition, Runx2 directs pluripotent MSCs to the osteoblast lineage and triggers the expression of major bone matrix protein genes (like type I collagen or *Alp*) in early progenitors. Then, FoxO1 interacts with Runx2 and could promote osteoblast differentiation [16,23]. Therefore, to some extent, FoxO1 promotes osteoblast differentiation by interacting with Runx2 or its gene promoter (Figure 2). Besides, FoxO1 could also bind to the ALP gene promoter [24]. BMP-2-induced FoxO1 transcription increases the reporter activity of an ALP gene promoter construct [25]. Thus, FoxO1 may also promote osteoblast differentiation by interacting with the ALP gene promoter (Figure 2).

Similarly, it has been reported that global deletion of FoxO1, 3, and 4 in mice leads to a reduction in the expression of Runx2, Osterix, and alkaline phosphatase in osteoblastic progenitors in vitro [26], and the mice also display a significant decrease in the number of cell colony-forming unit (CFU) fibroblasts and CFU osteoblasts presented in the bone marrow [21]. That means FoxOs could regulate osteoblast differentiation and proliferation.

On the other hand, FoxO1, as a transcriptional trans-repressor, inhibits the actions of peroxisome proliferator-activated receptor γ (PPARγ) [27], which activates adipogenesis and suppresses osteoblastogenesis. The deletion of FoxOs increases PPARγ expression [21]. Consistent with these findings, FoxO1 restrains adipogenesis by repressing the activity of the PPARγ promoter and by antagonizing PPARγ’s ability by directly binding to PPAR-response elements (PPREs), the promoters of PPARγ target genes in pre-adipocytes [28,29]. As a result, FoxOs improve bone formation, by increasing osteoblast differentiation at the expense of adipocyte differentiation from their common mesenchymal progenitors.

#### 3.1.2. FoxOs Repress Osteogenesis

In contrast to the above findings, some researchers also indicate FoxOs repress the osteogenic actions by suppressing Wnt signaling in osteoblast progenitors. Conditional knocked-out mice of FoxO1, 3, and 4 in bipotential progenitors of osteoblast and adipocytes showed that osteoblast number and bone mass were increased, but unexpectedly, there were no changes in the redox balance. The increased bone mass rooted in the increased proliferation of osteoprogenitor cells and bone formation, which accounted for the upregulation of Wnt/β-catenin signaling and cyclin D1 expression [18]. In addition, overexpression of FoxO1 reduced the MC3T3-E1 cell number and the number of proliferating cell nuclear antigen (PCNA)-positive cells with little effect on apoptosis, indicating that FoxO1 suppressed preosteoblast proliferation [25].

Moreover, in osteoblast progenitors, FoxOs acts as defense molecules in oxidative stress to regulate osteogenic actions. Excessive accumulation of reactive oxygen species (ROS) generally causes oxidative stress and destroys proteins, lipids, and DNA, even consequentially leading to cell death [30]. As a result of the conditional deletion of FoxO1, FoxO3, and FoxO4 in 3-month-old mice, oxidative stress and osteoblast apoptosis increased in bone, and the number of osteoblasts, the rate of bone formation, and bone mass were all decreased at cancellous or cortical sites [26]. So, we can infer that FoxOs can protect osteogenesis against oxidative stress. However, in osteoblast precursors, FoxOs are induced by the oxidative stress (exemplified by H_2_O_2_) to bind with β-catenin to co-activate the target genes’ transcriptions of FoxOs at the expense of Wnt/TCF-mediated transcription and osteoblast differentiation (Figure 2) [31]. Moreover, in aging mice, the expression of β-catenin/TCF-target genes decreases whereas FoxO-target genes increases in bone, along with an increase in markers of oxidative stress and a decrease in bone formation [31,32]. In line with this idea, fatty acid-binding protein 4 (FABP4)-Wnt10b mice, which express the Wnt10b transgene in marrow, and the mice with a mutation (G171V) in the low-density lipoprotein receptor related protein 5 (LRP5), both reveal enhanced bone mass and no evidence of age-related loss of bone mass or strength [21,33,34].

Similarly, the lipid oxidation may also suppress Wnt signaling by the same mechanism, and result in the decline in osteoblast number and bone formation that occurs with aging. It has been shown that lipoxygenases oxidize polyunsaturated fatty acids to form products, which bind to and activate PPARγ and generate pro-oxidants like 4-hydroxynonenal (4-HNE) [35]. The Almeida group demonstrated that lipid oxidation increases with age in bone [21]. Similar to H_2_O_2_, the 4-HNE also activates FoxOs that in turn attenuate β-catenin/TCF-mediated transcription [36]. Hence, it also enhances PPARγ levels for the suppression of TCF-mediated transcription to PPARγ expression [37,38]. On the other hand, the PPARγ bind to β-catenin and induce β-catenin degradation [36,39], thereby further diminishing β-catenin/TCF-mediated transcription. Therefore, at least in part, it is reasonable to understand that the bone loss with aging might arise from the repressive effect of ROS on Wnt/β-catenin signaling via FoxO activation.

Responding to oxidative stress, FoxOs also restrain other main osteogenic signaling pathways (Hedgehog signaling and Mitogen-activated protein kinases signaling) while boosting adipogenic signaling pathways [40]. Meanwhile, they also promote expression of antioxidants, which also increases adipogenic differentiation [40]. It is known that adipogenic differentiation is antagonistic to the osteogenic differentiation. Thus, FoxOs may also accelerate bone loss with aging by enhancing adipogenic differentiation. Obviously, in response to oxidative stress, FoxOs play an essential role to maintain skeletal homeostasis in osteoprogenitor cells.

Taken together, in osteoblast progenitor cells, FoxOs have complicated and contradictory features (Figure 2). There is no doubt that FoxOs are important in defense during oxidative stress and prevent cell death triggered by ROS, thus promoting osteogenesis. On one hand, MSCs have relatively high expression of antioxidant-like glycolysis and low ROS levels to keep quiescent [40], but a high ROS level may act as an intracellular signal to drive MSCs to exit from quiescence and result in the activation of a genetic program triggering osteoblast precursors commitment [16]. So, at the initial stage of differentiation, the switching from FoxO-mediated transcription to Wnt/TCF-mediated transcription may benefit osteogenesis. On the other hand, with the progress of osteogenic differentiation, FoxO1 may directly participate in osteoblastogenesis promotion by binding to the ALP and Runx2 gene promoters or directly interacting with Runx2, as well as maintaining a low level of ROS to protect cells from oxidative stress. Besides, FoxOs decrease ROS levels to restrain adipogenic differentiation. Therefore, the specific role of FoxOs may depend on the differentiation stage and ROS level of osteoprogenitor cells. Further studies are still needed to fully reveal the multiple functions of FoxOs in osteoprogenitor cells at a subtle stage during osteogenic differentiation.

### 3.2. FoxOs Improve Osteoblasts Function

**Figure 3:**
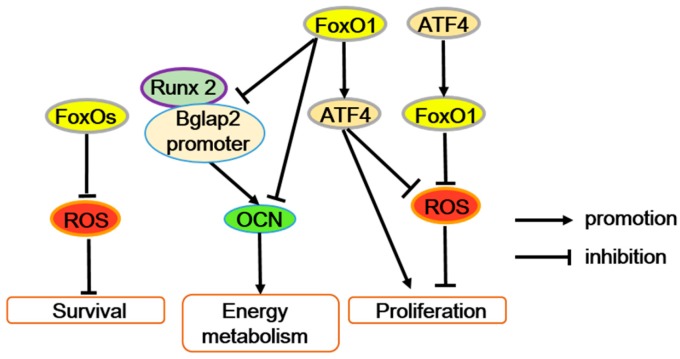
FoxOs’ function in osteoblasts. FoxOs’ transcriptional activity reduces ROS levels and improves osteoblast survival. The interaction between FoxO1 and activating transcription factor 4 (ATF4) maintains osteoblast normal proliferation by preventing ROS or enhancing protein synthesis. In addition, FoxO1 suppresses the interaction between the Runx2 and *Bglap2* (osteocalcin gene) promoters, directly bind to the Bglap2 promote r, or both to inhibit osteocalcin (OCN) expression.

The effects of FoxOs on osteoblasts are also complicated and likely to depend upon specific conditions (Table 2). Nevertheless, most of the literature support the positive effect of FoxOs in osteoblasts. On one hand, FoxOs regulate osteoblast numbers significantly by improving antioxidant defense (Figure 3). Conditionally overexpressed FoxO3 in osteoblasts decreases ROS and further inhibits osteoblast apoptosis with reducing phosphorylation of p66Shc. Consistent with this, conditional deletion of all three FoxO members (FoxO1/3/4) in 3-month-old mice decrease osteoblast numbers due to osteoblast apoptosis with arising oxidative stress. FoxOs inactivation also inhibits osteoblastogenesis [26]. Moreover, osteoblast-specific deletion of FoxO1 leads to increased oxidative stress, which decreases osteoblast numbers and bone mass [41]. More precisely, the FoxO1 inactivation weakens its interaction with activating transcription factor 4 (ATF4), reducing the target gene binding activity of FoxO1 and impeding amino acid import and protein synthesis through ATF4 (Figure 3). Therefore, as transcriptional regulation genes of FoxO1, antioxidant gene (such as *Sod2* and catalase) expression decreases, increasing the ROS level and exerting harmful effects on osteoblast survival. And with FoxO1 deletion, the blocked protein synthesis breaks the redox balance by improving the expression of p19ARF (an alternate reading frame protein product of the CDKN2A locus in mice) and p16 and downstream activation of their target protein p53, repressing the cell cycle of the osteoblasts and finally decreasing osteoblast numbers. Additionally, the blocked protein synthesis also suppresses glutathione accumulation, which induces oxidative stress [41]. Taken together, FoxOs enhance the activity of osteoblasts and protect these cells through the induction of antioxidants (Figure 3).

However, FoxO1 can restrain the energy metabolism function of osteoblasts through inhibiting osteocalcin (OCN) expression. Furthermore, FoxO1 can suppress the interaction between the Runx2 and *Bglap2* (OCN gene) promoters, directly bind to the *Bglap2* promoter, or both to reduce the transcriptional activity of *Bglap2* [16,42,43,44]. Generally, OCN also improves bone mineralization, calcium ion homeostasis, and prevents excessive mineralization. Thus, it is possible that FoxO1 mediates the dual roles of OCN in osteogenesis. But, obviously the effect of FoxO1 on Runx2 activity in osteoblasts is opposite in mesenchymal progenitors, and the mechanism is not well known. In addition, accompanied by immune response, FoxO1 also potentially contributes to immune-mediated inhibition of bone formation through accelerating osteoblast apoptosis [45].

Therefore, among the three FoxO proteins, FoxO1 is mainly required for redox balance and proliferation of osteoblasts and thereby to regulate bone formation.

### 3.3. FoxOs Positively Regulate Hematopoietic Stem Cell Activities

**Figure 4:**
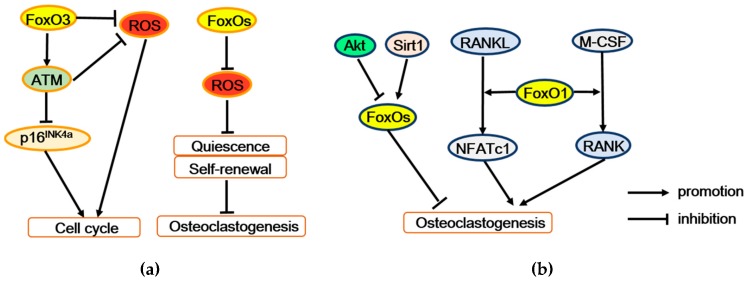
FoxOs regulate osteoclastogenesis and bone resorption. (**a**) FoxOs promote survival and self-renewal of hematopoietic stem cells by enhancing the expression of antioxidant enzymes. Moreover, FoxO3 also regulates expression of the ATM tumor suppressor to decrease ROS level and p16INK4a expression, thereby maintaining cell cycling and self-renewal. (**b**) FoxO1 stimulates osteoclastogenesis may by mediating the effect of M-CSF or RANKL on osteoclast precursors. But conversely, FoxOs restrain osteoclastogenesis, which mediated the regulation of Akt or Sirt1. ATM = ataxia telangiectasia mutated, p16INK4a = p16 (also known as multiple tumor suppressor 1), Akt = Protein kinase B, Sirt1 = Sirtuin 1, RANK = receptor activator of the NF-κB, RANKL = receptor activator of the NF-κB ligand, M-CSF = macrophage colony-stimulating factor 1, NFATc1 = nuclear factor of activated T cells 1.

Hematopoietic stem cell (HSC) is an osteoclast progenitor. FoxOs maintain quiescence and self-renewal of HSCs mainly through transcriptional regulation of cell cycle arrest and oxidative stress resistance (Figure 4a). The loss of FoxO3 in HSCs not only leads to oxidative DNA damage in HSCs by interfering in the base excision repair pathway [46], but also damages the HSC pool with regulating the cell cycle through ROS-independent modulations of the tumor suppressor protein ataxia telangiectasia mutated (ATM) and p16INK4a and ROS-mediated activation of p19ARF/p53/p21CIP1/WAF1/Sdi1 tumor suppressor pathways [47]. Likewise, conditional deletion of FoxO1, FoxO3, and FoxO4 in the adult mice hematopoietic system decreases the expression of antioxidant enzyme genes, including catalase, glutathione peroxidase 1 (*Gpx-1*), and superoxide dismutase *Sod1*, *Sod2*, and *Sod3*, resulting in ROS over-accumulation. The following physiologic oxidative stress increases HSCs’ apoptosis and drives HSCs out of quiescence into the cell cycle. Meanwhile, HSCs repopulating activity have been long-term impaired. All of these imply that FoxOs deficiency enforces HSCs into terminal differentiation at the expense of self-renewal. Finally, combined FoxOs’ deletion markedly decreases HSCs’ population and also expanses myeloid lineage [48]. Therefore, FoxOs are likely to serve as protectors of HSCs, decreasing the number of osteoclast progenitor cells to some degree (Table 3).

### 3.4. FoxOs Regulate Osteoclastogenesis

#### 3.4.1. FoxOs Activate Osteoclastogenesis

Complex osteoclastogenesis is governed by macrophage colony-stimulating factor 1 (M-CSF) and receptor activator of the NF-κB (RANK) ligand (RANKL), as well as other cytokines secreted by osteoblasts and osteocytes that control various steps of the osteoclast differentiation process, including precursor proliferation, commitment, differentiation, and maturation [49]. In osteoclast precursors, M-CSF can stimulate expression of RANK, which promotes osteoclast differentiation [50]. FoxO1 deletion in bone marrow macrophages (BMMs) decreases M-CSF-induced RANK expression and migration of osteoclast precursors [51]. RANKL indirectly induces nuclear factor of activated T cells 1 (NFATc1) of osteoclast precursors, dramatically facilitating osteoclast differentiation [52]. FoxO1 deletion in osteoclast precursors reduces expression and nuclear localization of NFATc1 [51]. In line with this, conditional deletion of FoxO1, FoxO3, and FoxO4 in 3-month-old mice reduces the expression of osteoclastogenesis related factors like the calcitonin receptor, tartrate-resistant acid phosphatase (TRAP), and cathepsin K [26]. Therefore, FoxOs should activate osteoclastogenesis by mediating the effect of M-CSF or RANKL on osteoclast precursors (Figure 4b).

#### 3.4.2. FoxOs Suppress Osteoclastogenesis

Other researchers have demonstrated that FoxOs negatively mediate RANKL-induced osteoclast formation. Overexpressing FoxO3 in monocyte/macrophage lineage cells decreases several resorption markers and increases bone mass [21]. The phosphoinositide 3-kinase (PI3K)/Akt pathway is the main negative regulator of FoxOs activity [6]. In osteoclast precursors, RANKL activates Akt signaling (like Akt/PI3K signaling) to promote proliferation, differentiation, and also attenuate apoptosis [50,53,54]. Along with these evidence, the deletion of FoxO1, FoxO3, and FoxO4 or overexpression of FoxO3 or mitochondria-targeted catalase in osteoclast precursors further elucidate that FoxOs upregulate the H_2_O_2_-inactivating enzyme catalase, and attenuates H_2_O_2_ accumulation, partly restraining the RANKL–Akt mediated osteoclastogenesis action [55]. Besides, Tan et al. have indicated that FoxO1 antagonizes ROS generation and thereby represses Akt, mitogen-activated protein kinases (MAPKs), and nuclear factor kappa-B (NF-κB), including the activated pathways of RANKL-induced osteoclastogenesis [56]. What is more, the inhibitory effect of FoxO1 on osteoclastogenesis is partially mediated by suppression of transcription factor MYC [57], which has been shown to reduce ROS production by inhibiting mitochondrial function [4,58,59]. Taken together, phosphorylation and inhibition of FoxO activity might partially mediate the positive effects of RANKL-induced Akt in osteoclast precursors (Figure 4b).

Beyond that, RANKL also prevents FoxO activity via acetylation. But Sirtuin 1 (Sirt1) deacetylates FoxO1, FoxO3, and FoxO4, and thereby stimulates FoxO-mediated transcription of catalase and hemeoxygenase-1 (HO-1). In osteoclast precursors, HO-1 attenuates mitochondrial oxidative phosphorylation and ATP production [60] and, along with catalase, reduce H_2_O_2_ levels. Both low ATP and H_2_O_2_ levels repress osteoclastogenesis [61]. Therefore, Sirt1 contributes to the anti-osteoclastogenic effects of FoxOs (Figure 4b). Similarly, mammalian sterile 20-like (Mst) kinase 1 (Mst1) is also a FoxO activating kinase and might facilitate osteoclast apoptosis via activation of FoxO-mediated transcription. When osteoclasts apoptosis is induced by serum withdrawal, staurosporine or bisphosphonates, Mst1 is identified as a key intermediate [62]. In detail, these apoptosis-inducing treatments promote the caspase cleavage of Mst1 into a positive 34 kDa species that keeps the catalytic domain and conduces to phosphorylate FoxOs [63]. However, it remains unknown whether FoxOs mediate the actions of Mst1 on osteoclast generation and survival.

#### 3.4.3. FoxOs, Expressed in Osteoblasts, Indirectly Repress Osteoclastogenesis

Unexpectedly, FoxOs also repress osteoclastogenesis indirectly via osteoblasts. In mice, conditional deletion of FoxO1 or overexpression of FoxO3 in osteoblasts results in increased or reduced osteoclast numbers, respectively [26]. Hence, activated FoxOs in osteoblastic cells also reduce osteoclast numbers. Osteoprotegerin (OPG), largely produced by osteoblasts, can bind and neutralize RANKL and block osteoclast formation in vitro and bone resorption in vivo [64]. Targeted deletion of FoxO1 in osteoblasts in mice diminishes OPG expression in bone. And FoxO1 activated or not in osteoblasts, in vitro, increases or decreases OPG, respectively [42]. Therefore, FoxO1 may control osteoclast numbers by increasing the OPG expression of osteoblasts. However, mice with overexpressed FoxO3 in osteoblasts does not exhibit any altering OPG levels in bone, hence indicating that FoxO3 might regulate osteoclast numbers independent of OPG [26].

Above all, in osteoclastogenesis, a relatively high ROS level increases HSC apoptosis and drives HSCs out of quiescence into the cell cycle and further terminal differentiation at the expense of self-renewal. But FoxOs fight against oxidative stress and thus protect HSCs from osteoclast differentiation. Moreover, in osteoclast precursor cells, relatively high ROS level promotes osteoclastogenesis [65]. But FoxOs also attenuate osteoclastogenesis or osteoclast activity largely for their oxidative resistance role, which is activated by Sirt1 or Mst1 and suppressed by Akt. In contrast, some researchers have suggested that FoxO1 stimulates osteoclastogenesis by mediating the effects of MSC-F or RANKL on osteoclast precursors. Nevertheless, ROS level is important for FoxOs for controlling osteoclastogenesis; the majority of the literature demonstrates that FoxOs negatively regulate osteoclastogenesis (Figure 4; Table 3 and Table 4).

### 3.5. FoxOs Facilitate Chondrogenesis

In human and mice models, FoxO1 and FoxO3 expressions are dramatically decreased in the superficial zone of cartilage regions during joint aging. There are obvious FoxO phosphorylation and cytoplasmic localization in chondrocyte clusters of osteoarthritis (OA) cartilage [66]. Furthermore, combined deletion of FoxO1, 3, and 4 in mice growth plate chondrocytes (Col2Cre-FoxO1, 3, and 4 triple knockout; Col2Cre-TKO) and Col2Cre-FoxO1 single knockout results in the overall body and tail length increasing, the articular cartilage becoming thicker in young mice, and severe skeletal deformities occurring at older stages, such as hyperkyphosis or OA-like changes in cartilage, synovium, and subchondral bone. These abnormalities are associated with the distinctly elongated hypertrophic zone of the growth plate or the increased height of the proliferative zone of the proximal tibial growth plate [67,68]. Noticeably, FoxOs are indispensable for the growth, development, and health of cartilage (Table 5).

**Figure 5:**
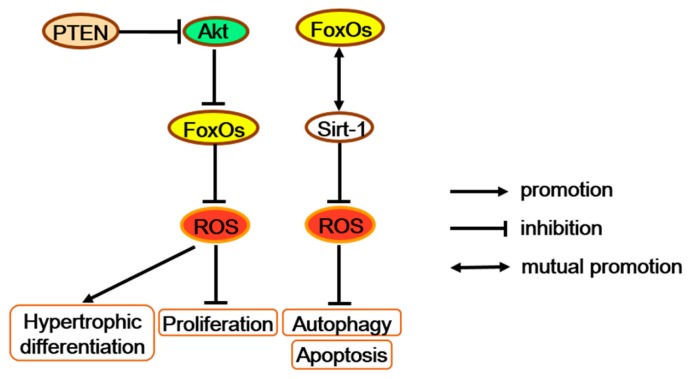
FoxOs increase antioxidants and further regulate multiple functions of chondrocytes. FoxOs possibly promote proliferation and block hypertrophic differentiation along with the PTEN/Akt/FoxO signaling. FoxOs also repress apoptosis and autophagy with altering the SIRT-1 protein level. PTEN = phosphatase and tensin homolog deleted on chromosome ten.

Chondrogenesis begins with the mesenchymal cells differentiating to chondrocytes, which then proliferate, hypertrophically differentiate, and terminally be apoptotic. FoxO3A expression is upregulated during chondrogenesis of human primary MSCs, blocking the hypertrophic differentiation and promoting apoptosis [69]. In human chondrocytes, downregulation of FoxO transcription factors with siFoxO1 and siFoxO3 reduces cell viability in response to ROS. The reduced antioxidant proteins, such as the ROS scavengers, GPX-1, and catalase, lead to an increase in intracellular oxidative stress. And the simultaneously raised autophagy-related proteins, such as microtubule-associated protein 1A/1B-light chain 3 (LC3) and Beclin1, give rise to ROS-induced apoptosis [70]. Furthermore, similar to the strongly reduced sirtuin 1 (SIRT-1) expression in OA cartilage [71], FoxO1 or FoxO1+3 (FoxO1 and FoxO3) downregulation also suppresses SIRT-1; this regulates oxidative stress and autophagy by modulating the signaling of FoxO and p53 [72], improves human chondrocytes survival via reducing proapoptotic protein tyrosine phosphatase 1B (PTP1B) [73], and also manages autophagy directly via deacetylation of key components (such as ATG5, 7, and 8) in the autophagy induction network [74]. Besides, the decreased SIRT-1, in response to siFoxO, brought about reduced FoxO transcriptional activity [70]. Collectively, in response to ROS in chondrocytes, FoxOs mediate antioxidant and autophagy proteins through altering the SIRT-1 level to some extent (Figure 5).

Combined deletion of FoxO1, 3, and 4 in mice growth plate chondrocytes (chondrocyte triple knock-out; CTKO) significantly reduces expression levels of oxidative stress defense genes, such as *Gpx3*, *Sepp1*, and *Sesn3*, in the primary chondrocytes, which may elevate the levels of ROS and promote more proliferated chondrocytes turning to pre-hypertrophic and hypertrophic chondrocytes [67,75]. Interestingly, chondrocyte-specific inactivation of the tumor suppressor gene phosphatase and tensin homolog deleted on chromosome ten (PTEN) also generated a disorganized neonatal growth plate, hyperkyphosis, and an increased total body length, which is similar to the phenotype of FoxOs CTKO mice [76]. Besides, PTEN can act as the upstream of FoxOs by regulating Akt activation [24]. Moreover, the Akt/FoxO pathway enhances chondrocyte proliferation but decreases chondrocyte maturation and cartilage matrix production [77]. Therefore, as antioxidant factors in chondrocytes, FoxOs may promote proliferation and block hypertrophic differentiation through the signaling along the PTEN/Akt/FoxO axis (Figure 5).

In addition, FoxO1 downregulation in chondrocytes also dramatically upregulates the expression of the inflammatory factor, chemerin, which increases chondrocyte apoptosis to some degree [70]. Through transcription factor FoxO1, pro-inflammatory cytokines TNF-α activates chondrocyte apoptosis and accelerates the loss of cartilage in diabetic fracture healing [78,79]. Moreover, TNF-α promote the chemokine expression of chondrocytes in diabetic fracture healing [80]. Hence, FoxO1 may protect diabetic fracture healing from an inflammation effect through TNF-α/chemokine.

In short, by decreasing ROS accumulation during chondrogenesis, FoxOs promote proliferation and block hypertrophic differentiation along with the PTEN/Akt/FoxO signaling, as well as repress apoptosis and autophagy by altering the Sirt1 protein level. All of these demonstrate that FoxOs contribute to relieving osteoarthritis and improve the growth, development, and health of cartilage.

## 4. Conclusion and Perspective

In summary, through FoxO deletion or overexpression in early osteoblast progenitors, osteoblasts, hematopoietic stem cells, osteoclast precursors, or chondrocytes, these sufficient and seminal papers have revealed the complicated and prominent roles of FoxO transcription factors not only in regulating bone cells function, but also in controlling the bone modeling or remodeling and preserving skeletal homeostasis.

**Figure 6:**
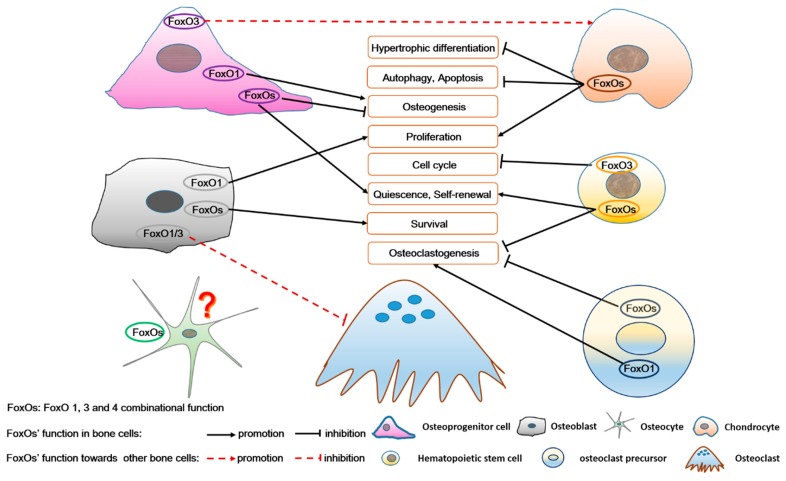
The role of FoxOs in osteoprogenitor cells, chondrocytes, osteoblasts, osteocytes, hematopoietic stem cells, and osteoclast precursors, as well as in the complex intercellular signaling among these bone cells. FoxOs not only regulate survival, self-renewal, cell cycle, proliferation, differentiation, autophagy, and apoptosis of bone cells, but also participate in the interaction among these bone cells. FoxO’s function in osteocytes is unknown.

In particular, FoxOs regulate bone cell survival, cell cycle, proliferation, differentiation, autophagy, even apoptosis, and also participate in network control among different kinds of bone cells (Figure 6). They are intimately involved in many bone physiological and pathological activities, such as bone development, bone remodeling, energy metabolism, aging, osteoarthritis, low bone mass in type 1 diabetes, postmenopausal osteoporosis, age-related bone loss, and myeloid leukemia. For example, FoxOs improve expression of antioxidant enzymes and maintain relatively low ROS levels to protect MSCs/HSCs from osteogenesis/osteoclastogenesis. During differentiation, ROS levels are increased due to enhanced cell metabolism. FoxOs promote osteogenesis and inhibit adipogenesis or osteoclastogenesis by decreasing ROS levels. Moreover, age-related mechanisms intrinsic to bone and oxidative stress are more proven as the protagonist of osteoporosis (including postmenopausal osteoporosis) [81]. Therefore, in bone, all the three FoxO members (FoxO1, 3, and 4) jointly mediate the redox balance of these bone cells, thereby regulating bone metabolism and also involved in osteoporosis pathology. Above all, these roles of FoxOs may help in the development of pharmacological agents, targeting FoxOs to further elucidate the treatment of bone metabolism diseases.

Before that, several questions should be paid attention to, including but not limited to the following: Firstly, how FoxO factors balance the positive and negative regulation of bone formation? On one hand, FoxO1 facilitates bone formation by interacting with Runx2 in osteoprogenitor cells or with ATF4 in osteoblasts, while diminishing bone formation by mediating the function of M-CSF/RANKL in osteoclasts. Here, FoxO1 should be an important factor for bone formation, but not involved in redox regulation. On the other hand, FoxOs mediate the redox balance in or among bone cells, thereby regulating bone metabolism and homeostasis. Therefore, the balance role of FoxOs may depend on the bone metabolism stage and bone redox homeostasis. Secondly, what are the complete and precise mechanisms of FoxOs performing functions among the different kinds of bone cells? For example, FoxOs regulate redox balance involved in the activities of all kinds of bone cells. Then, how the FoxOs mediate ROS signaling in or among bone cells? Thirdly, how FoxOs regulate bone remodeling and preserve skeletal homeostasis during aging in bone? For example, with aging, excessive osteocyte apoptosis correlates with oxidative stress and favors osteoclastogenesis, which leads to increased bone remodeling imbalance and bone loss. This results in the occurrence of several bone diseases, especially osteoporosis. Then, does FoxOs inhibit osteocyte apoptosis by regulating an anti-oxidation system in osteocytes? And what is the mechanism involved therein? Is it effective to treat and prevent bone loss? In light of this evidence, more research is necessary to further illuminate the precise role of FoxOs, especially for the development of new therapeutic approaches.

## Figures and Tables

**Table 1:** The bidirectional regulation role of FoxO transcription factors (FoxOs) in osteoprogenitor cells.

FoxOs	Effects on Bone	Functions in Osteoprogenitor Cell	Mechanisms	Cell/Mice Models	References
FoxO1	skeletogenesis (+); craniofacial development (+); craniofacial area (+)	osteogenesis differentiation (+); calcification culture (+)	FoxO1 interacts with *Runx2* promoter (+); Runx2, ALP and OCN expression (+)	C3H10T1/2 cells with FoxO1 overexpression or silencing; mice with downregulated FoxO1 expression in developing embryos in vivo/embryonic tibiae ex vivo	[22]
		osteoblast differentiation (+); osteoblast proliferation (-)		depletion/overexpression of FoxO1 in MC3T3-E1 cells	[25]
FoxO1, 3, and 4	oxidative stress (-); skeletal homeostasis (+)	CFU-osteoblasts (+);		conditional deletion of FoxO1, 3, and 4 in 3-month-old mice	[21]
		osteogenic differentiation (+)	Runx2, Osterix, and ALP expression (+)		[26]
		Adipogenesis (-)	PPARγ (-)	global deletion of FoxO1, 3, and 4 in mice	[21]
	bone mass (-); osteoblast numbers (-)	osteoprogenitor cells proliferation (-)	Wnt/β-catenin signaling (-); cyclin D1 expression (-)	mice lacking FoxO1, 3, and 4 in bipotential progenitors of osteoblast and adipocytes (expressing Osterix1)	[18]

Note: (+) refers to a positive effect and (-) refers to a negative effect.

**Table 2:** Bone metabolism regulatory functions of FoxO transcription factors in osteoblast.

FoxOs	Effects on Bone	Functions in Osteoblast	Mechanisms	Cell/Mice Models	References
FoxO1, 3, and 4	bone mass (+); bone formation rate (BFR) (+)	osteoblast number (+); osteoblast apoptosis (-); oxidative stress (-)	osteoblast number (+) through osteoblastogenesis (+); osteoblast apoptosis (-) through a cell-autonomous mechanism that enhances oxidative stress	conditional deletion of FoxO1, 3, and 4 in 3-month-old mice	[26]
FoxO1	BFR (+); bone volume (+)	osteoblast numbers (+); oxidative stress (-)	ROS activates the p53 signaling cascade, inducing cell cycle arrest and limiting osteoblast proliferation.	FoxOs deletion mice in bone	
FoxO3	vertebral bone mass (+); BFR (+)	osteoblast number (+), osteoblast apoptosis (-), oxidative stress (-)	ROS (-); phosphorylation of p66 ^Shc^ (-)	mice overexpressing FoxO3 under the control of the osteocalcin promoter	
FoxO1	bone mass (+), BFR (+) bone volume (+)	osteoblast proliferation (+), oxidative stress (-)	FoxO1 interacts with ATF4 and promotes amino acid import to favor the protein synthesis, such as glutathione. FoxO1 reduces ROS, activating a p53 signaling cascade, then promoting cell cycle.	FoxO1 deletion in mice from collagen1a1 expressing cells	[41]

**Table 3:** Bone metabolism regulatory functions of FoxO transcription factors in hematopoietic stem cell.

FoxOs	Effects on Bone	Functions in Hematopoietic Stem Cell	Mechanisms	Cell/Mice Models	References
FoxO3		oxidative DNA damage in HSC and progenitor cell (-)	ROS (-); the base excision repair pathway (+)	mice model with FoxO3^−/−^ in HSC	[47]
FoxO3		HSC quiescence (+); HSC G2/M transition (-)	ROS-independent modulations of ATM and p16^INK4a^ and ROS-mediated activation of p53/p21^CIP1/WAF1/Sdi1^ tumor suppressor pathways (-)		[46]
FoxO3	bone mass (+)	osteoclast number (-)		mice overexpressing FoxO3 in monocyte/macrophage lineage cells	[21]
FoxO1, 3, and 4		HSC quiescence (+); HSC compartment survival (+)	partly by impairing detoxification of ROS, FoxOs decreases HSC apoptosis and HSC-specific entry into the S/G2/M and G1 phases of the cell cycle	conditional deletion of FoxO1, 3, and 4 in the adult mice hematopoietic system	[48]

**Table 4:** Bone metabolism regulatory functions of FoxO transcription factors in osteoclast and progenitor cells.

FoxOs	Effects on Bone	Functions in Osteoclast and Progenitor Cell	Mechanisms	Cell/Mice Models	References
FoxO1, 3, and 4	bone resorption (-)	osteoclast progenitor proliferation (-); osteoclast lifespan (-)	FoxOs upregulate the H_2_O_2_-inactivating enzyme catalase and attenuates H_2_O_2_ accumulation	FoxO1,3,4^f/f^; LysM-Cre C57BL/6 mice; transgenic C57BL/6 mice: mitochondria-targeted catalase in osteoclasts	[55]
FoxO1, 3, and 4	bone mass (+); BFR (+)	osteoclast progenitor numbers (-); osteoclast apoptosis (-)	FoxOs increase the expression of the osteoclast-specific markers like the calcitonin receptor, TRAP, and cathepsin K.	conditional deletion of FoxO1, 3, and 4 in 3-month-old mice	[26]
FoxO3	vertebral bone mass (+); BFR (+)	osteoclast progenitor numbers (-); osteoclast numbers (-)		mice overexpressing FoxO3 under the control of the osteocalcin promoter	
FoxO1	bone resorption (-); osteoclast surface (-)			FoxO1 deletion in mice from collagen1a1 expressing cells	[41]
FoxO1		osteoclast differentiation (-); osteoclast activity (-)	MAPKs, NF-κB and AP-1 (-); MYC activity (-); ROS (-);	bone marrow mononuclear cells; RAW264.7 cells	[57]
FoxO1	osteoclastogenesis of calvarial bone (+)	osteoclastogenesis (+); osteoclast activity (+); osteoclast precursor migration (+)	FoxO1 activates osteoclast formation by mediating the effect of RANKL on NFATc1 and several downstream effectors; FoxO1 deletion or knockdown reduces M-CSF induced RANK expression and migration of osteoclast precursors.	LyzM.Cre^+^FoxO1 ^L/L^ mice; BMMs or RAW264.7 cells transfected with FoxO1 siRNA	[51]

Note: TRAP = Tartrate-resistant acid phosphatase, AP-1 = activator protein 1, NFATc1 = nuclear factor of activated T cells 1.

**Table 5:** Bone metabolism regulatory functions of FoxO transcription factors in chondrocytes.

FoxOs	Effects on Bone	Functions in Chondrocyte	Mechanisms	Cell/Mice Models	References
FoxO1, FoxO3		chondrocyte viability (+); chondrocyte apoptosis (-)	FoxO1 and FoxO3 up-regulated antioxidant proteins and autophagy-related proteins, but decreased expression of ADAMTS-4 and chemerin.	human articular chondrocyte transfected into siFoxO1 and siFoxO3	[70]
FoxO1, 3, and 4	hypertrophic zone of the growth plate (-); overall body and tail length at eight weeks of age (-); hyperkyphosis (-)		expression of genes involved in redox homeostasis (+)	FoxO1,3a,4^f/f^; Collagen2-Cre mice;	[67]
FoxO1, 3, and 4	total body and tail length at 1 month of age (-); height of the proliferative zone of proximal tibial growth plate at P7 and 1 month (-); articular cartilage thicker at 1 or 2 months of age (-); OA-like changes developed in cartilage, synovium, and subchondral bone between 4 and 6 months of age (-)	chondrocyte proliferation (-); cell density (+);	autophagy and antioxidant defense genes (+); Prg4 expression (+)	Col2Cre-FoxO1, 3, and 4 triple knockout mice (Col2Cre-TKO); Col2Cre-FoxO1 knockout mice	[68]
FoxO3 or 4	no cartilage abnormalities until 18 months of age			Col2Cre-FoxO3 or 4 single knockout mice	
FoxO1, 3, and 4	spontaneous cartilage degradation and OA severity in a surgical model or treadmill running of skeletally mature mice (-)	cell density (+);	Prg4 expression (+)	deletion of FoxO1/3/4 in mature mice using Aggrecan-CreERT2	
FoxO3		cell apoptosis (+); chondrogenic differentiation (-)	expression level of markers specific for mature (aggrecan, collagen II) and hypertrophic (collagen X) chondrocytes (-)	multipotent mesenchymal stromal cells (MSCs)	[69]

## Abbreviations

FoxOs	Forkhead box class O family member proteins
LPS	Lipopolysaccharide
TNF-α	Tumor necrosis factor α
MSCs	Mesenchymal stem cells
Runx2	Runt-related transcription factor 2
ALP	Alkaline phosphatase
OCN	Osteocalcin
CFU	Colony forming unit
PPARγ	Peroxisome proliferator-activated receptor γ
PPREs	PPAR-response elements
PCNA	Proliferating cell nuclear antigen
ROS	Reactive oxygen species
FABP4	Fatty acid-binding protein 4
4-HNE	4-hydroxynonenal
ATF4	Activating transcription factor 4
BFR	Bone formation rate
HSC	Hematopoietic stem cell
Gpx-1	Glutathione peroxidase 1
M-CSF	Macrophage-colony stimulating factor
RANK	Receptor activator of NF-κ B
RANKL	Receptor activator of NF-κ B ligand
BMMCs	Bone marrow mononuclear cells
NFATc1	Nuclear factor of activated T cells 1
Akt	Protein kinase B
SIRT-1	Sirtuin 1
TRAP	Tartrate-resistant acid phosphatase
MAPKs	Mitogen-activated protein kinases
NF-κB	Nuclear factor kappa-B
HO-1	Hemeoxygenase-1
Mst 1	Mammalian Sterile 20-like kinase 1
OPG	Osteoprotegerin
AP-1	Activator protein 1
PI3K	phosphoinositide 3-kinase
OA	Osteoarthritis
PTP1B	Protein tyrosine phosphatase 1B
CTKO	Chondrocyte triple knock-out
PTEN	Phosphatase and tensin homolog deleted on chromosome ten
